# Rhizospheric microbial consortium of *Lilium lancifolium* Thunb. causes lily root rot under continuous cropping system

**DOI:** 10.3389/fmicb.2022.981615

**Published:** 2022-10-26

**Authors:** Liangliang Dai, Sunil K. Singh, Hao Gong, Yuanyuan Tang, Zhigang Peng, Jun Zhang, Dousheng Wu, Huiming Zhang, Danxia He

**Affiliations:** ^1^Changsha General Survey of Natural Resources Center, Changsha, China; ^2^Shanghai Center for Plant Stress Biology, CAS Center for Excellence in Molecular Plant Sciences, Chinese Academy of Sciences, Shanghai, China; ^3^College of Biology, Hunan University, Changsha, China; ^4^School of Biological Sciences, Nanyang Technological University, Singapore, Singapore

**Keywords:** rhizosphere, lily root rot disease, geological soil, continuous cropping, microbiota

## Abstract

Tiger lily (*Lilium lancifolium* Thunb.) is a cash crop with a long history of cultivation in China. Its roots have long been used as a valuable component of Chinese medicine. Continuous cropping, the conventional planting approach for tiger lily, often leads to severe root rot disease, but it is not yet clear how this planting method leads to root rot. In this study, we analyzed the rhizosphere microbiome and predicted microbial protein function in tiger lily planted with the continuous cropping method in three different geological types of soil. In order to explore the specific rhizosphere microbiota triggering root rot disease, tiger lily was compared to maize grown in a similar system, which showed no disease development. An analysis of the chemical elements in the soil revealed that the *Pseudomonas* and *Streptomyces* genera, with pathogenic functions, were dominant in the tiger lily rhizosphere. The lower soil pH of tiger lily compared to maize supports the accumulation of pathogenic bacteria in the tiger lily rhizosphere. Meanwhile, we discovered that bacteria of the *Flavobacterium* genus, with their predicted phosphate transport function, specifically accumulated in the maize rhizosphere. Our findings suggest that *Pseudomonas* and *Streptomyces* bacteria may result in continuous cropping–induced root rot disease in tiger lily and that *Flavobacterium* could serve to protect maize from pathogenic bacteria.

## Introduction

There is a close relationship between plants and the microbiota populating their roots and the surrounding soil, particularly the rhizosphere, where microbes transport nutrients released from the plant’s roots (Cheng et al., 2003; Igiehon and Babalola, 2018; Zhou et al., 2018; Zu et al., 2022). Rhizosphere microbes are capable of influencing the fitness of plants through multiple mechanisms, such as phosphate solubilization, which stimulates plant growth by increasing phosphate uptake from the soil (Alori et al., 2017), or through the production of pathogenic factors that cause disease symptoms (Kudjordjie et al., 2019; Kwasna et al., 2021). Over the past few decades, the interactions between rhizosphere bacteria and plants have become a key area of scientific investigation, in which bacteria characterized as beneficial are used as fertilizers to increase crop production or enhance their vitality and fitness (Dellagi et al., 2009; Beirinckx et al., 2020; Shang et al., 2020; Cadot et al., 2021; Ruger et al., 2021). The composition of the rhizospheric bacterial community is affected by changes in environmental factors such as soil humidity, soil pH, and organic compounds content, which differ across soil types (Cao et al., 2018; Lei et al., 2021; Khmelevtsova et al., 2022). In addition, the rhizospheric bacterial community is also affected by plant genotypes (Jamalzadeh et al., 2021; Kaushal et al., 2021; He et al., 2022), implying a degree of host selectivity on the establishment of these communities.

It is widely accepted that rhizospheric bacterial communities are closely associated with plant health. Beneficial rhizosphere bacteria antagonistically protect plants from infection by pathogens (Chi et al., 2019). However, soilborne pathogens in the rhizosphere microbiota can also cause yield loss. Previous studies have suggested that plants recruit beneficial bacteria to defend them from pathogens (Chi et al., 2019; Wu et al., 2020). For example, Dudenhöffer et al. (2016) demonstrated that barley plants recruit antifungal microbes in response to *Fusarium graminearum* attack by altering the composition of the rhizospheric bacterial community. Similarly, an abundance of bacteria from the *Acidobacteria* phylum and the *Pseudomonas* and *Chthonomonas* genera has been implicated in the suppression of fusarium wilt disease in banana (Shen et al., 2015). Therefore, understanding which rhizosphere bacteria influence plant health, and how, is of great interest.

Tiger lily, a well-known cash crop, is widely distributed throughout the world, and holds promising economic and medicinal value (Chi et al., 2019; Shang et al., 2020). However, the production of tiger lily is severely threatened by plant disease (Lawson, 2011; Shang et al., 2016). The factors underlying disease in tiger lily have been classified into three main categories: viruses, soilborne pathogenic fungi, and bacteria (Lawson, 2011). *Pseudomonas marginalis* and *Pectobacterium carotovorum* bacteria have been indicated as the chief causative agents of soft rot in tiger lily roots (Lawson, 2011).

Xiangxi prefecture is one of China’s key areas for lily plantation. Here, continuous cropping, the conventional planting approach for lilies, frequently induces root rot disease. An investigation of the rhizosphere microbiota revealed distinct differences between the microbial communities of healthy and diseased lily plants (Kande Vidanalage et al., 2016). Interestingly, continuous cropping of maize in Xiangxi does not cause disease in roots or other tissues. Moreover, maize fitness has been reported to be enhanced by endosphere bacteria under chilling conditions (Beirinckx et al., 2020). However, the effects of the maize rhizosphere microbiota composition on maize fitness are still elusive. Maize accounts for about 36% of China’s total national grain production (Cao et al., 2018). Its production is influenced by factors such as water, temperature, and soil nutrients, which affect the composition of microbes at the plant roots and in the rhizosphere (Cao et al., 2018; Kudjordjie et al., 2019). Therefore, investigating the tiger lily rhizosphere microbiome to identify its functional bacteria is an effective tool for reducing the symptoms of disease caused by continuous cropping.

For this study, we designed a 16S rRNA gene sequencing approach to illustrate the reasons for continuous cropping–induced root rot disease in tiger lily. In order to track the conserved bacterial communities, we collected tiger lily rhizosphere samples from Cambrian, Ordovician, and Silurian soil, which are the major geological soil types of China, from Xiangxi prefecture (Xu et al., 2000; Hongyu et al., 2017). Based on the Chinese Soil Taxonomy (CST) system (Cooperative Research Group on Chinese Soil Taxonomy, 2001), and considering factors such as soil texture, soil moisture, temperature class, ochric epipedon, argic horizon, and alic evidence, Cambrian, Ordovician and Silurian soils fall under the Argosols order (L). More precisely, Cambrian soil is grouped into the Carbonati-perudic Argosols (L3.1), and Ordovician and Silurian soil are grouped into the Ali-Udic Argosols (L3.2) and Hapli-perudic Argosols (L3.3), respectively.

To identify the rhizosphere microbial communities enriched specifically in continuous-cropped tiger lily, we investigated the maize rhizosphere in parallel, as this would reveal which symptoms are common to the continuous cropping method. Our results showed that the differential microbial assemblage at the lily root is the major cause of disease development in tiger lily, in contrast to other crops, such as maize, that are also cultivated under the continuous cropping system.

## Materials and methods

### Plant materials and growth conditions

Maize (*Zea mays* L., haideng 11) and tiger lily (*Lilium lancifolium* Thunb.) were obtained from Longyan Seeds Resource Center and Longyan Xiluo Supply and Marketing Cooperator, respectively. Maize seeds were stratified at 4°C for 48 h, then sterilized with 30% house bleach for 10 min, followed by five times washing in sterile double-distilled water. Tiger lily was directly cultured by bud cutting. The maize seeds (225 seedlings per area 5 × 5 m) and tiger lily bud cuttings (250 seedlings per area 5 × 5 m) were planted on Cambrian, Ordovician, and Silurian soil in Xiangxi prefecture (29.5° N, 109.5° E for maize; 29.4° N, 109.4° E for lily) under open natural conditions. Both the maize and tiger lily were planted continuously three times prior to sample collection; samples were collected after the third round of cultivation. After plantation in the soil, maize and tiger lily were grown continuously for 156 days (measuring stage), at which point the rhizosphere samples were collected for 16S rRNA gene sequencing. During growth, both maize and lily were cultivated with no fertilizers or other herbicides, and underwent weeding, plowing, and watering 2–3 times.

### Collection of rhizosphere samples

Microbiome samples were collected based on previous publications (Lareen et al., 2016; Kudjordjie et al., 2019; Kaushal et al., 2021). Briefly: the rhizosphere substrates were collected from maize and tiger lily grown on Cambrian, Ordovician, and Silurian soil in Xiangxi prefecture, Hunan province, China. Soil attached tightly to the plant roots was collected. Soil from the roots of 6 plants (chosen randomly from a 5 × 5 m^2^ area to avoid any bias caused by uneven soil conditions) grown on one soil type was collected as one biological replicate. Four biological replicates of each sample were prepared for DNA extraction. Detailed information on the samples is available in Supplementary Table 1A.

### Microbiome sample preparation and 16S rRNA gene sequencing

DNA sample preparation and 16S rRNA gene sequencing were performed as previously described (Kaushal et al., 2021), with minor modifications. Briefly, total DNA was extracted with the FastDNA^®^ SPIN Kit for Soil (MP Biomedicals, Solon, OH, USA) following the manufacturer’s instructions. Microbiome DNA samples were eluted in 100 μl nuclease-free water and DNA concentrations were determined by using Nanodrop 2000 (Thermo Fisher Scientific, Waltham, MA, United States). The amplicon libraries were generated following the protocol of the Illumina Novaseq System for 16S rRNA gene metagenomic sequencing library preparation. Two rounds of polymerase chain reaction (PCR) were performed with the KAPA HiFi HotStart ReadyMix. The first-round PCR primers 799F (5′-AACMGGATTAGATACCCKG-3′) and 1193R (5′-ACGTCATCCCCACCTTCC-3′), which span ∼400 bp of the hypervariable regions V5–V7 of the prokaryotic 16S rRNA gene, were extended to 799F–B and 1193R–B by adding bridging sequences (5′-ggagtgagtacggtgtgc-3′ and 5′-gagttggatgctggatgg-3′) at their 5′ ends to better facilitate the second round of PCR. For each biological replicate, three technical replicates were conducted to increase the final amount. The PCR was conducted on a 96-well plate with the KAPA HiFi HotStart ReadyMix by using 2 μl of 8 ng/μl adjusted DNA template in a total volume of 25 μl, with 1 μl of 5 μM of each primer.

The first PCR reaction was performed as follows: initial denaturing at 95°C for 3 min, followed by 30 cycles of 95°C for 30 s, 55°C for 30 s, 72°C for 30 s, and extension at 72°C for 5 min. Triplicate reactions of each sample were pooled and a 15 μl aliquot was loaded on a 1% agarose gel. The PCR primers 799F and 1193R produce a mitochondrial product at ∼800 bp and a bacterial amplicon at ∼400 bp. The ∼400 bp bands were extracted from the gel with a sharp scalpel and eluted from the agarose using the QIAquick Gel Extraction kit (Qiagen, Hilden, Germany) as per the manufacturer’s protocol. After purification and elution in 80 μl nuclease-free water, the concentration of the amplicon DNA of each sample was determined by using the dsDNA HS Assay Kit (12640ES60, YEASEN, China) on Qubit 2.0 (Thermo Fisher Scientific, Waltham, MA, United States). The first-round PCR products were further barcoded during the second-round PCR following the protocol of the Illumina Novaseq System for the 16S rRNA gene metagenomic sequencing library preparation.

The second PCR amplification was conducted on a 96-well plate in 20 μl volume containing 4 μg first-round PCR products, 20 nM unique barcode primers (see Supplementary Table 1A), and 200 nM indexed sequencing adaptor primers (see Supplementary Table 1B). The second PCR reaction was run under the following conditions: 95°C for 3 min, then 15 cycles of 95°C for 30 s, 55°C for 30 s, 72°C for 1 kb/min, and extension at 72°C for 5 min. The second-round PCR products were inspected on 1% agarose gel and purified as described above. The DNA concentrations of each sample were determined with Qubit 2.0 and all the samples with equal molar amount were mixed into one tube for microbiome sequencing. At least 25 ng of each sample was required for sequencing. Samples were sequenced at the Genomics Core Facility of Novogene in Tianjin, China.

### Microbiome data analysis

The 16S rRNA gene sequencing data analysis was performed as previously described (He et al., 2022). Raw Tags were first obtained by using FLASH software^1^ (Magoc and Salzberg, 2011). High-quality Clean Tags were then obtained using *fastp* with quality control. The Clean Tags were subsequently run through Usearch software to compare with the SILVA database (v. 138.1) to detect and remove chimeras (Bolyen et al., 2019). After the chimeras were removed, Effective Tags were collected and then denoised using the *DADA2* module in the QIIME2 software (Callahan et al., 2016). Sequences with an abundance value less than 5 were filtered out, thus obtaining the final amplicon sequence variants (ASVs). The ASVs were then compared to SILVA using the *classify-sklearn* module in QIIME2 to obtain the species information of each ASV. The subsequent analysis mainly used QIIME2 (Bolyen et al., 2019; Estaki et al., 2020). A minimum of five sequences per ASV in at least one sample was used to generate the ASVs table (see Supplementary Table 2A). The weighted UniFrac distance and principal coordinates analysis were performed by QIIME2, as were the ASVs’ relative abundance, alpha diversity, beta diversity, and Venn diagram analysis. The significant differences between samples were assessed by ANOVA-based statistics with multiple tests (*p*-value < 0.05).

### Protein function prediction analysis

The protein function prediction analysis was conducted using PICRUSt2 software with the KEGG Orthology (KO) metagenome database. Heatmaps were created using the iDEP.90 online tool.^2^

### Soil characteristic assay

The soil of the rhizosphere samples used for 16S rRNA gene sequencing was also used for soil characteristics identification. Elemental silver (Ag) was determined by spectral lines with different sensitivity of alternating current arc emission spectroscopy (ES). Mercury (Hg) was determined by atomic fluorescence spectroscopy (AFS 8500). Iron (Fe) and phosphorus (P) were analyzed by inductively coupled plasma optical emission spectroscopy (ICP-OES, ICAP7400). Nitrogen (N) was determined by elemental analyzer (EA 5100). The pH of different soil samples was determined by the ion-selective electrode (ISE, PerfectlON) method. Organic compounds were determined with the titration method of ferrous sulfate and potassium dichromate sulfuric acid.

### Statistical analysis

One-way ANOVA with Tukey’s multiple comparison test, the Kruskal–Wallis test, and an ANOSIM assay were used for the statistical analysis of data in this study. Four biological replicates of each sample, with a *p*-value ≤ 0.05, indicated statistical significance between different samples. Student’s *t*-test was used for statistical significance analysis of soil characteristics with six biological replicates of each sample.

## Results

### Assessment of rhizosphere microbial communities and richness

In order to explore the reasons for root rot in tiger lily caused by continuous cropping, we conducted 16S rRNA gene sequencing to identify the microbiota using six rhizosphere samples. We described them with the following terms: Clily (tiger lily rhizosphere samples from Cambrian soil); Cmaize (maize rhizosphere samples from Cambrian soil); Olily (tiger lily rhizosphere samples from Ordovician soil); Omaize (maize rhizosphere samples from Ordovician soil); Slily (tiger lily rhizosphere samples from Silurian soil); Smaize (maize rhizosphere samples from Silurian soil). Maize, a major crop cultivated under similar conditions as tiger lily but without suffering from the same root rot disease, was selected as a parallel plant to better explore which candidate pathogenic bacteria are specifically associated with tiger lily. The plants were grown in the fields of Xiangxi prefecture, as described in the Section “Materials and methods.”

The hypervariable regions of V5–V7 in the prokaryotic 16S rRNA gene were amplified for the preparation of sequencing libraries. A total of 10,554 unique amplicon species variates (ASVs) were identified from all samples (Supplementary Table 2A). After combining ASVs with the same taxonomic annotation, a total of 1,162 ASVs (see Supplementary Table 2B) were obtained. ASVs without species identification were clustered into the Other category. A total of 235 unique species were identified (see Supplementary Table 2C).

**FIGURE 1:**
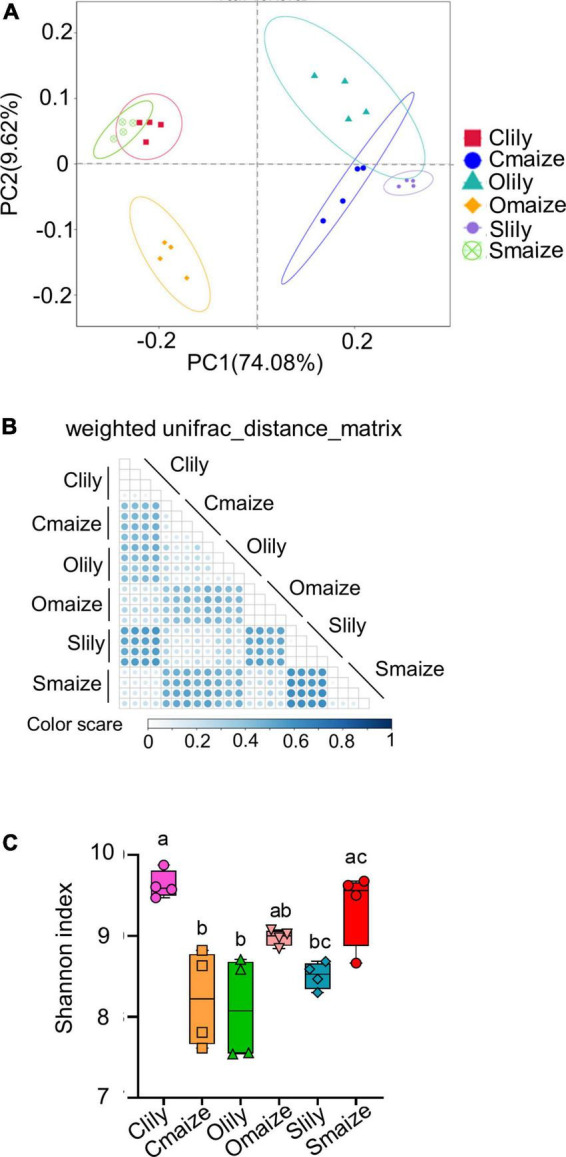
Geological soil type and plant species are the main factors underlying distinctive variations within rhizosphere bacterial communities. **(A)** Principal coordinate analysis (PCoA) of the bacterial communities of rhizosphere soil in maize and tiger lily, based on weighted unifrac metrics. *n* = 4 biological replicates. Clily = tiger lily rhizosphere samples from Cambrian soil; Cmaize = maize rhizosphere samples from Cambrian soil; Olily = tiger lily rhizosphere samples from Ordovician soil; Omaize = maize rhizosphere samples from Ordovician soil; Slily = tiger lily rhizosphere samples from Silurian soil; Smaize = maize rhizosphere samples from Silurian soil. **(B)** Heatmap of weighted Unifrac distance metrics, used to measure the dissimilarity coefficient between samples. The color scale indicates the difference in species diversity between paired samples; the smaller the value, the smaller the difference. **(C)** Shannon index of the rhizospheric bacterial communities represents the communities’ diversity in each sample. Means ± SE, *n* = 4. One-way ANOVA with multiple comparison tests was used for statistical analysis; different letter indicates statistical difference, *p* ≤ 0.05.

Sequence quality was measured by the paired-sequence combination rate with a minimum of 16 bp overlapped length among samples. More than 82% of the sequences of each sample were highly qualified (see Supplementary Figure 1A). Principal coordinate analysis (PCoA) of the ASVs showed obvious segregation of bacterial communities between the maize and tiger lily rhizospheres in Cambrian, Ordovician, and Silurian soil, with variation rates of 74.08% at the PC1 coordinate and 9.62% at the PC2 coordinate (Figure 1A). We further analyzed the bacterial diversity of different samples using the weighted UniFrac distance matrix to obtain more insights into the effects of soil type and crop species on bacterial communities (Figure 1B). Similarly to the PCoA results, the maize and tiger lily rhizospheric bacterial communities were found to be distinct from each other.

We also performed an alpha diversity assay in order to investigate the taxonomic diversity in different samples. A boxplot of the alpha diversity indicated significant differences in the distance between samples (see Supplementary Figure 1B). The total number of observed ASVs in each sample indicated that Clily possessed highly diverse communities compared with other samples (Supplementary Figure 1C). Similarly, the Shannon index (Figure 1C) also supports the finding that the rhizosphere microbiome in Clily is more dynamic compared with other samples.

### Bacterial community composition

**FIGURE 2:**
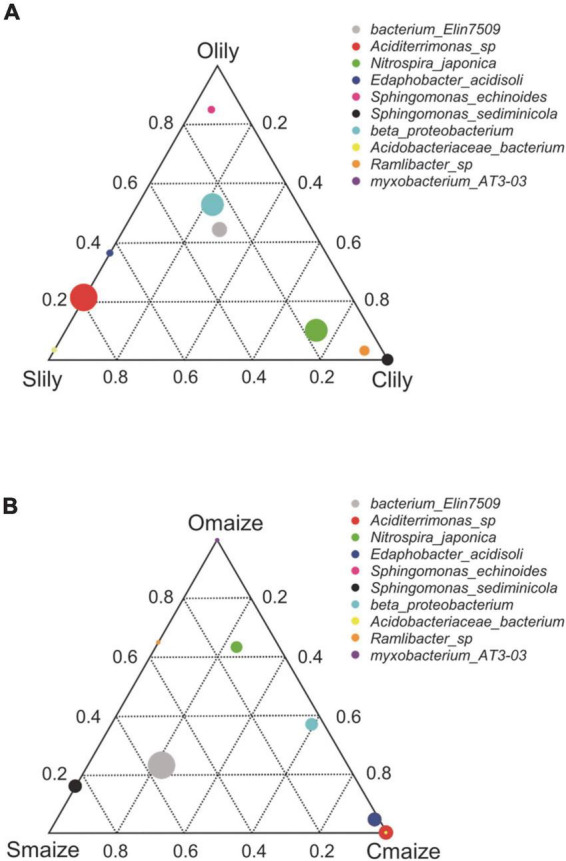
Cambrian soil shows more flexible bacteria community remodeling compared to Ordovician and Silurian soils. **(A)** Ternary plot of the top 10 abundant genera of the tiger lily rhizosphere across different soil types. *n* = 4 biological replicates. **(B)** Ternary plot of the top 10 abundant genera in the maize rhizosphere across different soil types. *n* = 4 biological replicates. Different dominant species of three samples at genus level. Vertex means samples. Circles represent species, and the relative abundance of each species is indicated by the size of the circle. The distance of the circles to vertexes presents the relative content of species in the sample.

**FIGURE 3:**
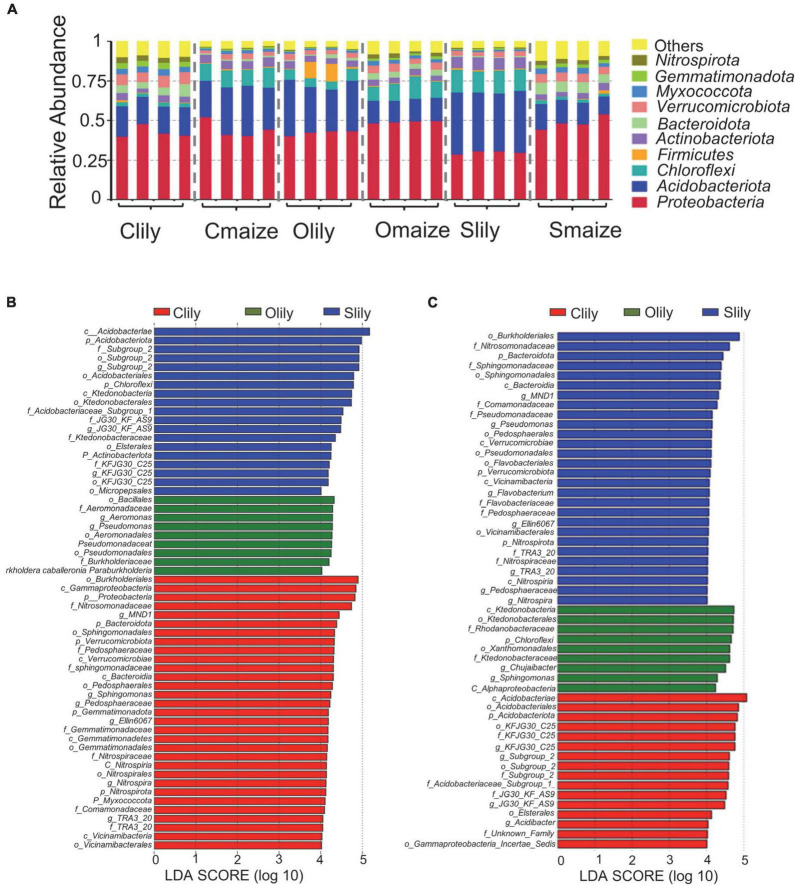
The rhizosphere bacterial community structure presents intrinsic bias according to crop species and soil type. **(A)** Taxonomic diversity of the top 10 phyla in different samples. *n* = 4 biological replicates. **(B)** Histogram of LDA scores of 16S rRNA sequences of tiger lily rhizosphere microbiome. **(C)** Histogram of LDA scores of 16S rRNA sequences of maize rhizosphere microbiome. The LDA value distribution histogram shows species whose LDA score is greater than 4, indicating a biomarker with statistical difference between the groups. The length of the histogram represents the impact size of the different species.

To further understand the differences between the bacterial communities in Clily, Cmaize, Olily, Omaize, Slily, and Smaize, we performed a ternary plot analysis from the phylum to the species level using identified microbiota from all samples (see Figure 2 and Supplementary Figures 2A–D). The top 10 abundant species in the tiger lily ternary plot showed *Sediminicola* as the most abundant species in Clily (Figure 2A). An analysis of the dominant species of maize in different soil types shows that *Aciditerrimonas* sp., *Acidobacteriaceae* bacterium, and *Edaphobacter acidisoli* were the dominant species in Cmaize and *Myxobacterium* AT3-03 was the most abundant species in Omaize (Figure 2B). The ternary plot of the top 10 phyla and families in tiger lily (Supplementary Figures 2A,B) and maize (Supplementary Figures 2C,D) showed a similar pattern, revealing that Cambrian soil exhibits the most dynamic bacterial communities compared with Silurian soil or Ordovician soil. We next analyzed the taxonomic structure of the ASVs at the phylum level using relative abundance (RA), which was measured by dividing the reads of ASV in a given sample to the total useable reads in that sample (Supplementary Table 2C). *Proteobacteria* and *Acidobacteriota* were the most dominant phyla (Figure 3A). Among the top 10 abundant phyla, *Nitrospirota*, a phylum containing many species of bacteria identified as beneficial to the plant (Umezawa et al., 2020), shows 2.7% RA occupation in Smaize, but only 0.8% in Slily (Figure 3A and Supplementary Table 2E). *Nitrospirota* occupied 2.2 and 0.5% in Omaize and Slily, respectively. However, Clily had an RA occupation of *Nitrospirota* of 3.3%, while that of Cmaize was only 0.5% (Figure 3A and Supplementary Table 2E). A linear discriminant analysis effect size (LEfSe) analysis indicated that the *Acidobacteriales*, *Pseudomonadales*, and *Burkholderiales* orders are dominant in Slily, Olily, and Clily, respectively (Figure 3B). *Burkholderiales*, *Ktedonobacterales*, and *Acidobacteriales* orders are the marker bacteria of Smaize, Omaize, and Cmaize, respectively (Figure 3C).

An analysis of the phyla with statistically significant alterations by tiger lily relative to maize (*p* < 0.05) among different soil types (Supplementary Figure 3 and Supplementary Table 2F) showed 12 shared phyla (Supplementary Figure 3C), along with 3 unique phyla in Cambrian and Silurian soils and 1 phylum in Ordovician soil (Supplementary Figure 3B). The RA occupations of the *Nitrospirota*, *Bacteroidota*, and *Gemmatimonadota* phyla are significantly higher relative to the other 9 shared phyla (Supplementary Figure 3C). Six of the phyla present significantly repressed RA in maize as compared with tiger lily (Supplementary Figure 3B). Taken together, these results illustrate that rhizospheric bacterial communities are shaped by crop species and soil types.

### Predicted function of the rhizosphere microbiota and soil characteristics

The above rhizosphere microbiota assay demonstrated that the microbial communities of maize and tiger lily were intrinsically different across all three soil types. We next predicted the function of the rhizosphere microbiota by using PICRUSt2 software based on the KEGG Orthology (KO) database. Principal component analysis (PCA) of the bacterial function KO terms showed a clear separation between maize and tiger lily among different soil types (Supplementary Figure 4A). We next investigated the conserved KOs altered by maize and tiger lily in the three soil types (Supplementary Figures 4B–D). We found 229 KO terms that were uniquely altered by tiger lily, and another 154 KOs that were uniquely altered by maize (Supplementary Figure 4D and Supplementary Table 2G).

**FIGURE 4:**
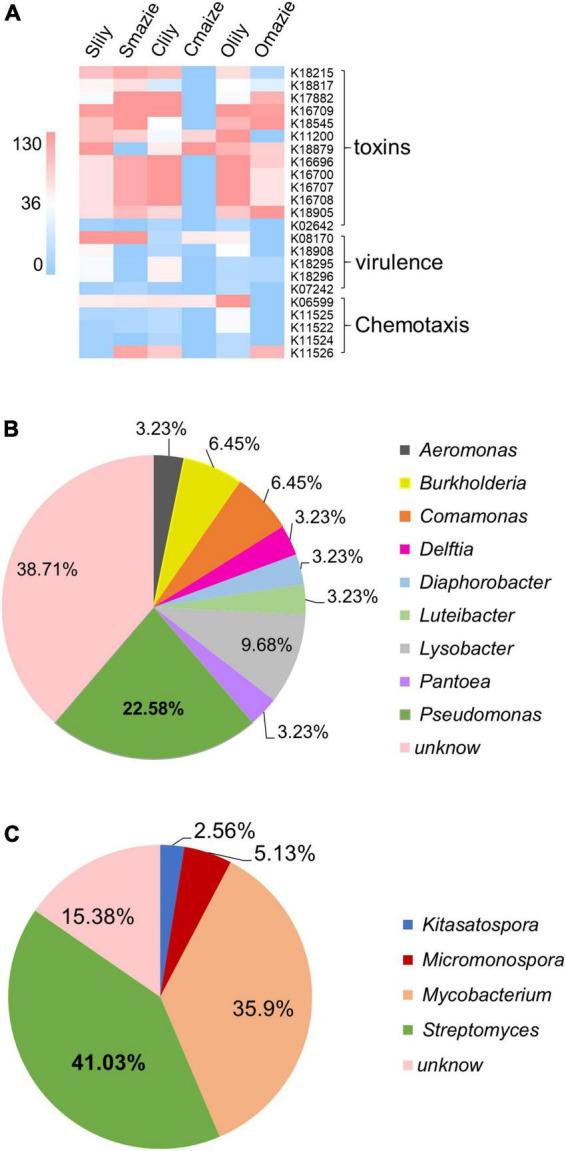
Microbial predicted function of the tiger lily rhizosphere microbiome using the KEGG Orthology (KO) database. **(A)** Heatmap of conserved virulent activities (23 Kos) of bacterial communities in tiger lily rhizosphere within the three different soil types. Color scale indicates bacterial abundance. **(B)** Distribution of the bacteria of 23 KOs at genus level within the *Gammaproteobacteria* class. **(C)** Distribution of the bacteria of 23 KOs at genus level within the *Actinobacteria* phylum.

Remarkably, among the 229 KOs specifically induced by tiger lily (Supplementary Table 2G), 23 KO terms belonging to pathogenic functions were uncovered (Figure 4A and Supplementary Table 2H). Moreover, we demonstrated that 40% of the *Proteobacteria* phyla and 30% of the *Actinobacteriota* phyla make up the main bacterial community among the 23 KOs [for *Proteobacteria*, 59.6% of the bacteria are of the *Gammaproteobacteria* class (Supplementary Table 2J)]. We also found that 22.58% of the *Gammaproteobacteria* were of the *Pseudomonas* genus (Figure 4B and Supplementary Figure 5B), which are recognized as major phytopathogens (Xin et al., 2018; Yadav et al., 2020). Similarly, 41.03% of the *Actinobacteriota* bacteria were of the *Streptomyces* genus (Figure 4C and Supplementary Figure 5C), which contains phytopathogens like *Streptomyces stelliscabiei* (Handique et al., 2021). These results suggest that the rhizospheric microbiome of the tiger lily is dominated by the *Pseudomonas* and *Streptomyces* genera, and, as these have a pathogenic function within the microbiome, they may be the cause of the root rot disease observed under the continuous cropping method.

Out of the 154 KOs influenced by maize, 8 KOs related to “phosphate transport” were dominant in Smaize, accounting for 45% of RA occupation (Supplementary Figure 5D and Supplementary Table 2H). We found that 86.7% of the bacteria with “phosphate transport” function were of the *Bacteroidota* phylum and 54.2% of the *Bacteroidota* we observed belong to the *Flavobacterium* genus (Supplementary Figure 5E), most of which has been reported as beneficial to plants (Soltani et al., 2010). Interestingly, the abundance of all these Flavobacteria (except ASV331) was higher in Smaize relative to Omaize and Cmaize (Supplementary Table 2L). Meanwhile, the RA of the *Nitrospirota* phylum was higher in Smaize compared with Omaize and Cmaize (Figure 3A). Taken together, this suggests that maize may utilize the beneficial functions of Flavobacteria to compete antagonistically with pathogenic bacteria and thereby get rid of continuous cropping–induced root rot disease. More importantly, these results indicate that Silurian soil may be an optimal soil for maize plants compared to Cambrian and Ordovician soils.

We also performed a statistical assay of the soil characteristics, namely, the key elements and pH (Table 1 and Supplementary Table 3), to investigate the effects of the rhizosphere microbiota on disease symptoms in tiger lily. No obvious differences were observed in terms of heavy metals among the different samples. Similarly, the concentrations of nitrogen, phosphorous, and organic compounds content showed no significant difference across the six samples. However, Clily showed a higher pH value compared to Slily. The pH of tiger lily–grown soils was relatively lower compared to maize-grown soils, suggesting that pathogenic bacteria grow more readily in tiger lily–cultivated soil because the acidic conditions are preferable for them (Xu et al., 2000; Ramos-Morales, 2012). Taken together, these results support the possibility that the overwhelming abundance of *Pseudomonas* and *Streptomyces*, genera with a pathogenic effect on tiger lily, results from acidic soil conditions.

**TABLE 1:** Statistical table of soil characteristics, including chemical elements and organic matter, in XiangXi perfecture.

Items	Ag	Hg	TFe_2_O_3_	N	P	Organic compounds	pH
	μ g/g	μ g/g	%	μ g/g	μ g/g	%	NA
Slily	0.039333 ± **0.005**	0.049 ± **0.005**	5.695 ± **0.25**	1157.167 ± **44.2**	736.6667 ± **49.1**	1.68 ± **0.103**	4.631667 ± **0.038**
Smazie	0.045 ± **0.005**	0.057667 ± **0.009**	4.91 ± **0.4**	1202 ± **89.5**	683.8333 ± **50.1**	1.831667 ± **0.190**	5.818333 ± **0.693**
Olily	0.05383 ± **0.008**	0.092 ± **0.02**	4.428333 ± **0.38**	1183.333 ± **87.3**	694 ± **121.2**	1.965 ± **0.165**	5.356667 ± **0.441**
Omazie	0.049167 ± **0.005**	0.066667 ± **0.01**	4.593333 ± **0.24**	1269.333 ± **35.3**	633.5 ± **24.9**	1.931667 ± **0.06**	6.213333 ± **0.434**
Clily	0.060167 ± **0.007**	0.206667 ± **0.02**	5.291667 ± **0.25**	1142.833 ± **63.6**	878.3333 ± **77.4**	1.856667 ± **0.11**	6.353333 ± **0.460**
Cmaize	0.073 ± **0.01**	0.149167 ± **0.03**	4.818333 ± **0.12**	1253.667 ± **125.5**	787.3333 ± **99.9**	1.99 ± **0.306**	7.26 ± **0.445**

Mean ± SE; *n* = 6 biological replicates. Student’s *t*-test was used for statistical analysis. No significant difference between tiger lily and maize is evident. Bold values are SE.

## Discussion

Studies have shown that the composition and diversity of plant rhizosphere microbial communities varies depending on plant species. However, these studies were conducted at plant seedling stage or with samples that did not undergo multiple rounds of plantation, and their analysis lacks a microbial function interpretation (Zhou et al., 2018). In this study, we performed a rhizosphere microbiota assay by using approximately 4-month-old tiger lily and maize with 3 rounds of plantation. We further analyzed the alterations in bacterial protein function induced by tiger lily and maize, with the goal of enhancing the understanding of microbial interference in the development of root rot disease in tiger lily triggered by continuous cropping.

We initially hypothesized that tiger lily contains a different rhizospheric bacterial community relative to maize under similar continuous cropping systems, and that this was the cause of the observed root rot disease. PCoA analysis indicates the existence of intrinsic rhizospheric bacterial communities in maize and tiger lily. In accordance with earlier studies, the *Proteobacteria* and *Acidobacteriota* phyla were dominant in all samples (Segata et al., 2011; Lareen et al., 2016; Igiehon and Babalola, 2018; Zu et al., 2022), while Cambrian soil showed a more flexible assemblage of bacterial communities relative to Ordovician and Silurian soils in both maize and tiger lily. The *Nitrospiraceae* family was recognized as one of the major bacteria in Smaize microbial communities, while the *Pseudomonas* genus was found to be dominant in the tiger lily rhizosphere microbiome. Identification of the soil characteristics showed that the pH of Clily is significantly higher than that of Slily, and that tiger lily–grown soil generally had a lower pH than maize-grown soil; Silurian soil therefore possesses more acidic (i.e., more preferable) conditions for pathogenic bacteria (Li et al., 2017), which potentially poses a threat to tiger lily health.

Predictions of the protein function of the rhizosphere microbiota showed that pathogenesis-related functional items were specifically clustered into 229 lily-altered KOs. A total of 9% (22.5% × 40%; proportion of class level and proportion of phylum level) and 12% (41% × 30%; proportion of genus level and proportion of phylum level), respectively, of the ASVs of pathogenesis-related functional terms were *Pseudomonas* and *Streptomyces*. These results indicate that the high abundance of *Pseudomonas* and *Streptomyces* bacteria may result in root rot disease in tiger lily grown under continuous cropping conditions. Notably, 56 of the 229 KOs altered by tiger lily were clustered into the photosynthesis function (Supplementary Table 2H). However, the reasons for the abundance of enriched bacteria with photosynthesis function in the tiger lily rhizosphere are unclear. One possibility is enriched *Chloroflexi* and *Acidobacteriota* phyla. A taxonomic diversity assay at the phylum level and a LEfSe assay showed that *Chloroflexi* and *Acidobacteriota* were major phyla in the tiger lily rhizosphere (Figure 3A). They have also been reported as key bacteria containing bacteriochlorophyll for photosynthesis (Abadi et al., 2020).

Lim et al. (2003) reported that *Fusarium oxysporum* caused lily root disease through vascular wilt. Although Hahm et al. (2003) indicated that *P. marginalis* was one of the reasons for soft rot disease in lily, the supporting evidence is limited. In this study, we explored various genera by using rhizomicrobiota and predicted protein function, and we found *Pseudomonas* and *Streptomyces* to be the major candidates likely responsible for root rot disease. However, the specific pathogenic strains accumulated in the tiger lily rhizosphere, and their pathogenic mechanisms, are not clear.

Synthetic microbial communities (SynCom) has been reported as a highly reproducible tool for investigating the relationship between plants and bacteria (Shayanthan et al., 2022). Follow-up studies will aim to isolate bacteria from infected lily grown under the continuous cropping system in order to detect specific strains and their pathogenic characteristics (Yadav et al., 2020). Synthetic microbial communities will be produced using isolated pathogenic strains, and lilies will be inoculated with them, for the purpose of confirming whether the candidate bacteria are responsible for root rot disease. The potential to generate a synthetic pathogenic bacterial community and conduct a deeper investigation of its mechanisms based on our investigation in this study is promising.

Our findings also reveal that *Flavobacterium* mainly accumulates in the maize rhizosphere, relative to that of tiger lily. Functional prediction of the maize microbiome indicated that *Flavobacterium* has a phosphate transport ability. As has been shown previously, most of the *Flavobacterium* genus can be used as plant growth–promoting bacteria thanks to their properties of phosphate solubilization and auxin and siderophore production (Soltani et al., 2010). A dominant abundance of beneficial bacteria in the rhizosphere will antagonize pathogenic bacteria (Yin et al., 2021), which may protect maize from disease caused by continuous cropping. The abundance of ASVs in the *Flavobacterium* genus is markedly higher in Silurian soil compared to Cambrian and Ordovician soils, indicating that maize may achieve higher biomass in Silurian soil. However, from a practical standpoint, crop biomass is affected by environmental factors apart from rhizosphere microbiota, and there is no clear record of differences in maize biomass among the three geological soils studied here.

## Conclusion

In conclusion, the composition of the rhizosphere microbiome plays important roles in crop cultivation and disease monitoring. We demonstrated the intrinsic differences in the rhizosphere microbiota between tiger lily and maize in three geological soil types: Ordovician, Silurian, and Cambrian soil. Our work revealed that the *Flavobacterium* genus, with its phosphate transport function, is the major beneficial genus for the growth of healthy maize in Silurian soil. *Pseudomonas* and *Streptomyces* bacteria, with their pathogenic function enriched in the tiger lily rhizosphere, may result in root rot disease under the continuous cropping method.

## Data availability statement

The datasets presented in this study can be found in online repositories. The 16S rRNA gene sequencing data is available in the NCBI SRA under BioProject PRJNA816815, and BioSample ID: SAMN26733807.

## Author contributions

SS and DW edited the languages and corrected typos. All authors contributed to the article and approved the submitted version.

## Footnotes

1 http://ccb.jhu.edu/software/FLASH/, v.1.2.11.

2
http://bioinformatics.sdstate.edu/idep/

## Supplementary material

Supplementary Figure 1 Plant species and soil type affects the microbial taxonomy of the plant rhizosphere (cf. Figure 1). **(A)** Sequencing quality of each sample. PE250 two-paired sequence assembly with overlapped sequence more than 16 bp. More than 82% of the sequence in each sample is high-qualified. One-way ANOVA was used for statistical analysis; *n* = 4 biological replicates. The presence of same letter above each bar indicates no significant difference between each group. **(B)** Alpha Diversity Index between-group difference boxplot. The horizontal axis of the boxplot represents the grouping, and the vertical axis represents the corresponding alpha diversity index value. In the table are the Kruskal–Wallis test results for all groups, or between two groups. Different letters indicate a significant difference. **(C)** Observed amplicon sequence variants (ASVs) of each sample. One-way ANOVA was used for statistical analysis; *n* = 4 biological replicates. Different letters indicate a significant difference.

Supplementary Figure 2 More flexible bacterial communities are reshaped in Cambrian soil compared to Ordovician and Silurian soils (cf. Figure 2). Ternary plot assay of the tiger lily rhizosphere microbiome in different soil types, at the phylum level **(A)** and family level **(B)**. Ternary plot assay of the maize rhizosphere microbiome in different soil types, at the phylum level **(C)** and family level **(D)**.

Supplementary Figure 3 Significant differences between tiger lily and maize rhizosphere phylain different soils (cf. Figure 3). **(A)** Shared and unique phyla of significantly different bacteria between tiger lily and maize rhizospheres in three soil types. **(B)** Relative abundance of unique phyla in each soil type. **(C)** The 12 conserved phyla of maize and tiger lily rhizosphere bacterial communities in Cambrian, Ordovician, and Silurian soils. Means ± SD, *n* = 4. Relative abundance was estimated by the abundance of amplicon sequence variant (ASV) in one sample, divided by the amount of ASVs in all samples.

Supplementary Figure 4 Overall profile of the predicted functions of tiger lily- and maize-changed bacterial communities (cf. Figure 4). **(A)** Principal component analysis (PCA) assay of predicted microbial function indicates that the functions of maize and tiger lily bacteria communities are separate from each other across the three geological soil types. KEGG Orthology database was used for the microbial function prediction. *n* = 4 biological replicates. **(B)** Shared and uniquely tiger lily–associated ASV KO items among all soil types. **(C)** Shared and uniquely maize-associated ASV KO items among all soil types. **(D)** Shared and unique KEGG Orthology (KO) items of tiger lily and maize.

Supplementary Figure 5 *Pseudomonas* and *Streptomyces* are the most abundant genera in bacterial communities with pathogenic function associated with tiger lily (cf. Figure 4). **(A)** Distribution of the bacteria of 23 KEGG Orthologys (KOs) at the phylum level. **(B)** Percentage relative abundance (RA) of the *Pseudomonas* genus within the *Gammaproteobacteria* class. The number in the bar panel is the amplicon sequence variant (ASV) number of *Pseudomonas.* **(C)** Percentage RA of the *Streptomyces* genus within the *Actinobacteria* phyla. The number in the bar panel is the ASV number of *Streptomyces.* **(D)** All conserved phosphate transport function categories across the different samples. Relative RA area indicates the relative occupation of each sample compared to all samples. The larger the area of a sample, the stronger the phosphate transport ability in that sample. **(E)** Distribution of the bacteria of 8 KOs at the phylum level. The bar stack indicates the proportion of bacteria at the genus level within the *Bacteroidota* phylum.

Supplementary Table 1 Samples information for microbiota. **(A)** Sample information, including sample ID, replication, primers, and locations. **(B)** Primers for the 16S library system.

Supplementary Table 2 Microbiota analysis results. **(A)** Total amplicon sequence variants (ASVs) that can be detected. **(B)** ASVs after combining ASVs with the same taxonomic annotation. **(C)** Relative abundance (RA) of ASVs with specific aligned species. **(D)** Shannon index and observed ASVs. **(E)** RA occupation at the phylum and family levels. **(F)** Significant unique and shared phyla, in tiger lily and maize, in all soil types. **(G)** Unique KEGG Orthologys (KOs) altered by maize and tiger lily. **(H)** Three key clusters of KOs (data for Figure 4 and Supplementary Figure 5). **(I)** ASVs with their related KO functions. **(J)** ASVs with tiger lily–specific predicted pathogenic function. **(K)** ASVs with maize-specific predicted “phosphate transport” function. **(L)** Abundance of restricted ASVs belonging to *Flavobacterium*.

Supplementary Table 3 Soil characteristics of the three geological soil types.
