# Supplementary figures and images for: Rhizospheric microbial consortium of Lilium lancifolium Thunb. causes lily root rot under continuous cropping system
Source: Front Microbiol. 2022 Oct 26;13:981615. doi: 10.3389/fmicb.2022.981615 (PMC9645529; doi:10.3389/fmicb.2022.981615)

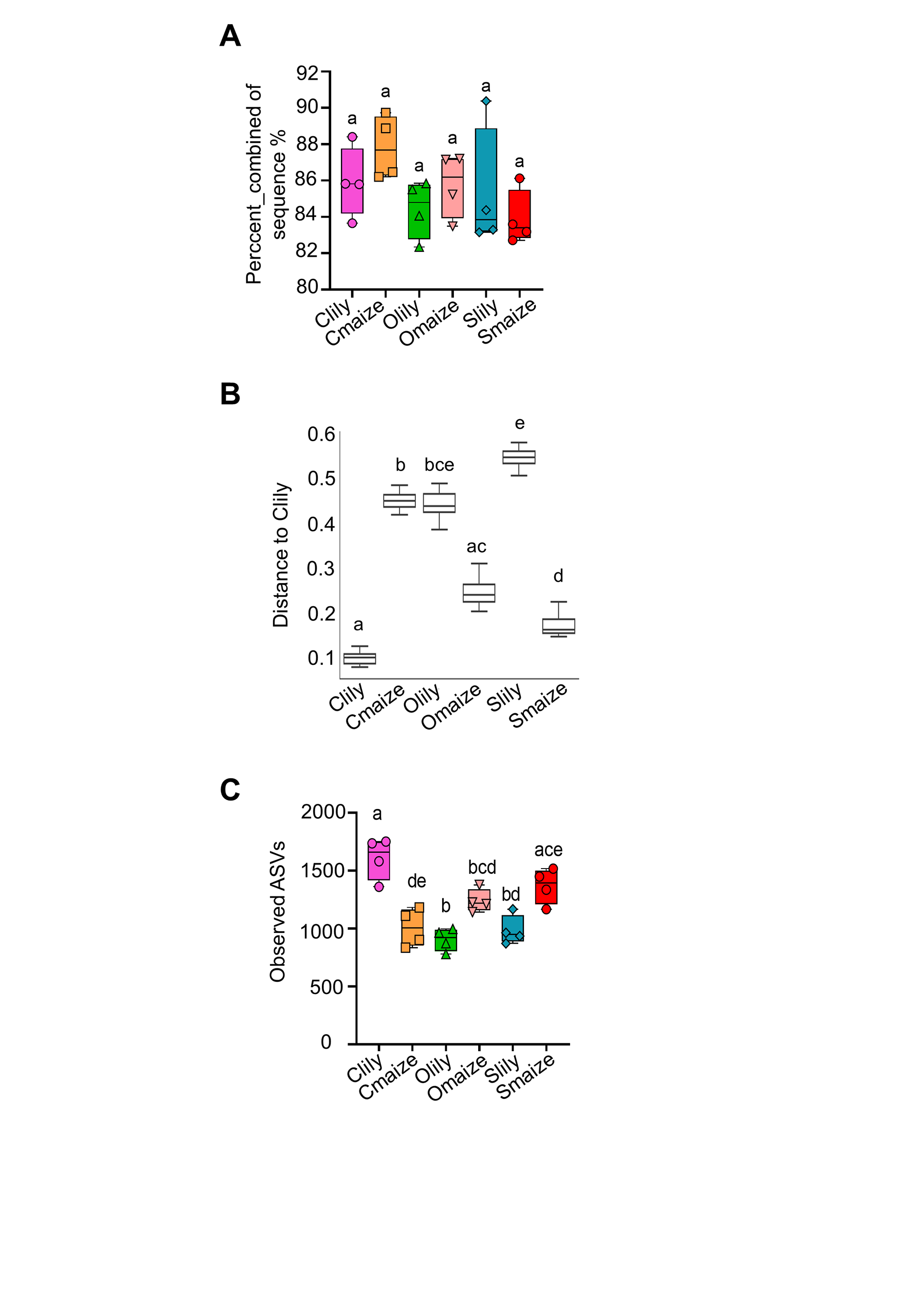

Supplement: Supplementary Figure 1 — Plant species and soil type affects the microbial taxonomy of the plant rhizosphere (cf. Figure 1). (A) Sequencing quality of each sample. PE250 two-paired sequence assembly with overlapped sequence more than 16 bp. More than 82% of the sequence in each sample is high-qualified. One-way ANOVA was used for statistical analysis; n = 4 biological replicates. The presence of same letter above each bar indicates no significant difference between each group. (B) Alpha Diversity Index between-group difference boxplot. The horizontal axis of the boxplot represents the grouping, and the vertical axis represents the corresponding alpha diversity index value. In the table are the Kruskal–Wallis test results for all groups, or between two groups. Different letters indicate a significant difference. (C) Observed amplicon sequence variants (ASVs) of each sample. One-way ANOVA was used for statistical analysis; n = 4 biological replicates. Different letters indicate a significant difference. [file Image_1.tif]

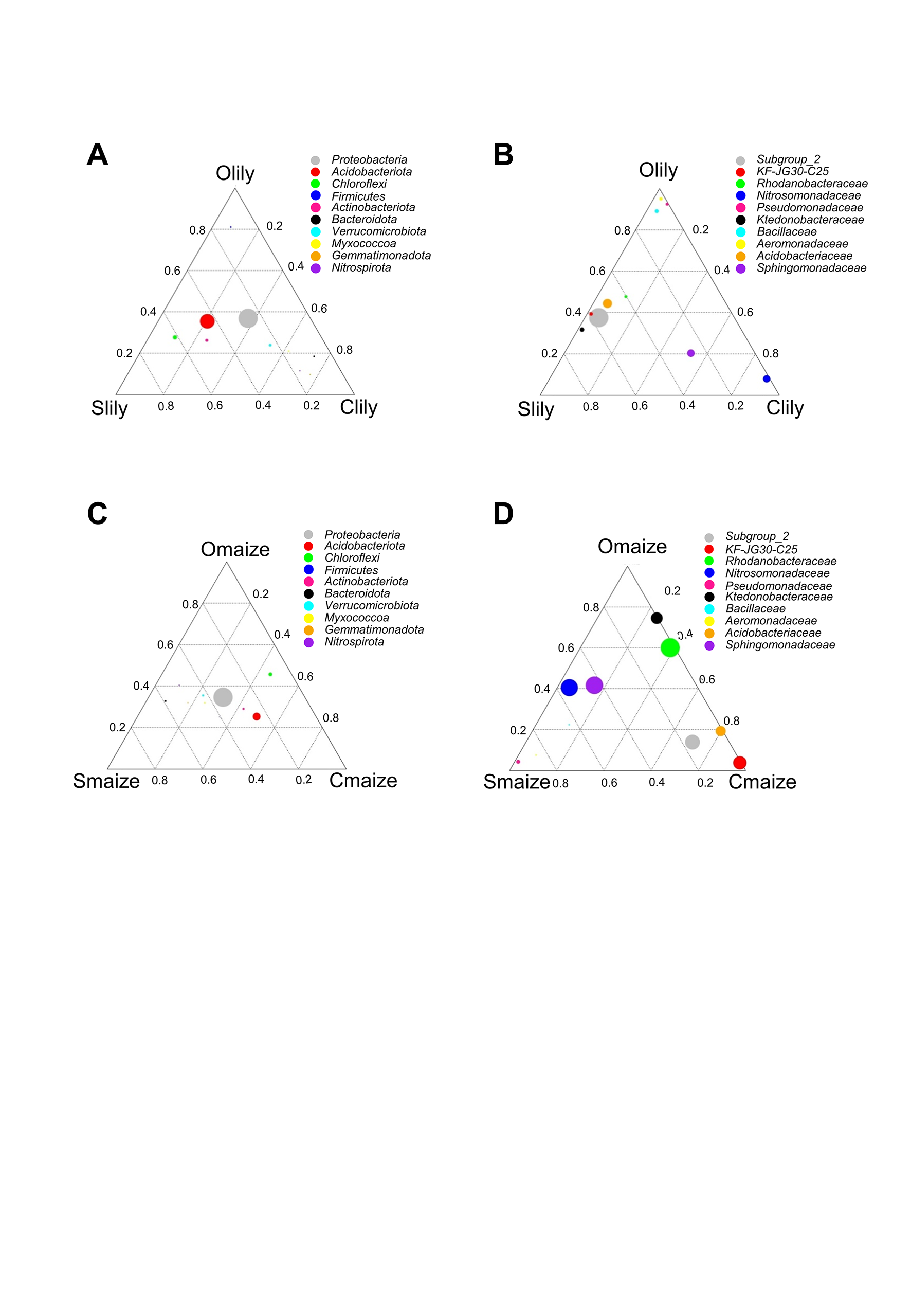

Supplement: Supplementary Figure 2 — More flexible bacterial communities are reshaped in Cambrian soil compared to Ordovician and Silurian soils (cf. Figure 2). Ternary plot assay of the tiger lily rhizosphere microbiome in different soil types, at the phylum level (A) and family level (B). Ternary plot assay of the maize rhizosphere microbiome in different soil types, at the phylum level (C) and family level (D). [file Image_2.tif]

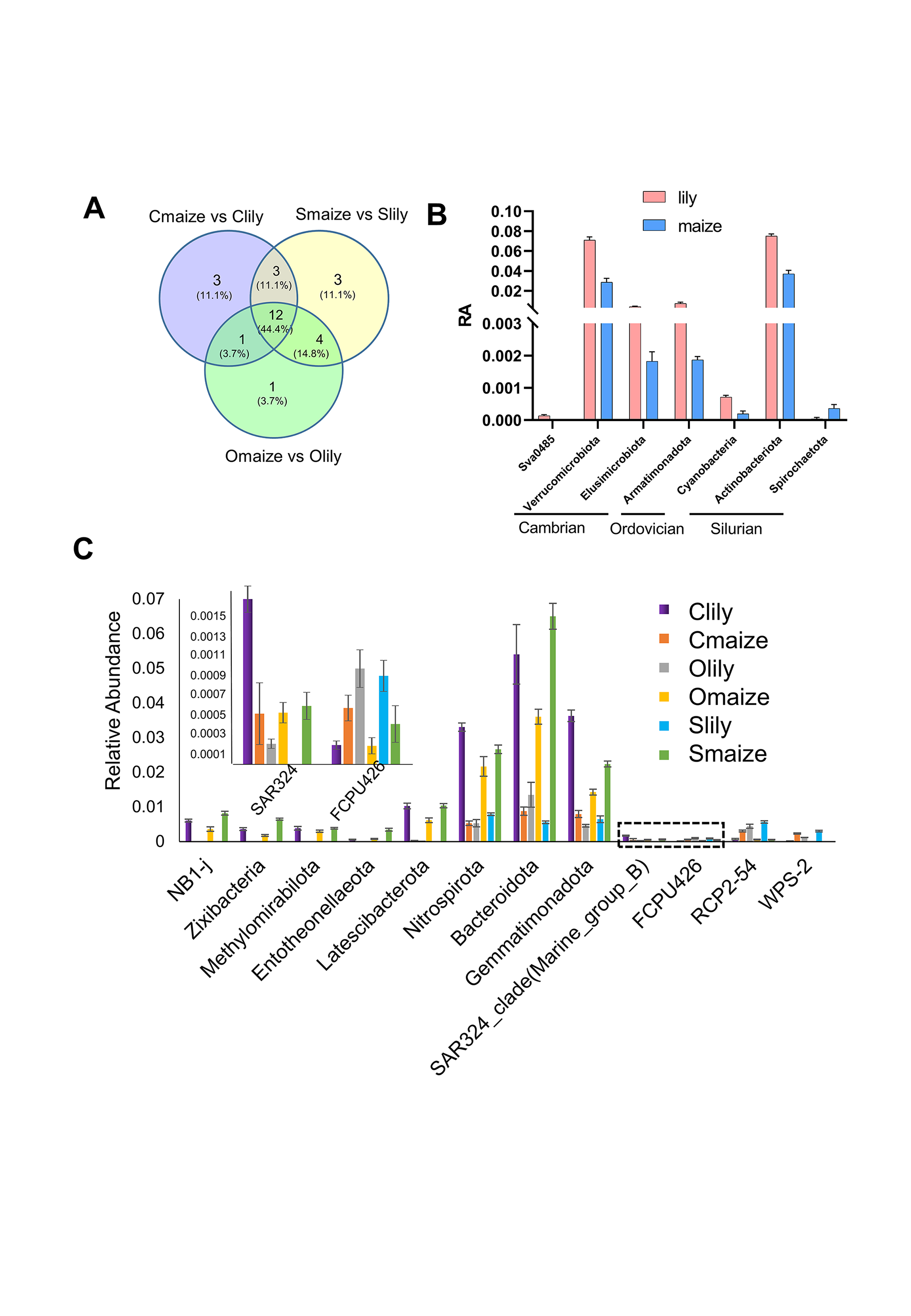

Supplement: Supplementary Figure 3 — Significant differences between tiger lily and maize rhizosphere phylain different soils (cf. Figure 3). (A) Shared and unique phyla of significantly different bacteria between tiger lily and maize rhizospheres in three soil types. (B) Relative abundance of unique phyla in each soil type. (C) The 12 conserved phyla of maize and tiger lily rhizosphere bacterial communities in Cambrian, Ordovician, and Silurian soils. Means ± SD, n = 4. Relative abundance was estimated by the abundance of amplicon sequence variant (ASV) in one sample, divided by the amount of ASVs in all samples. [file Image_3.tif]

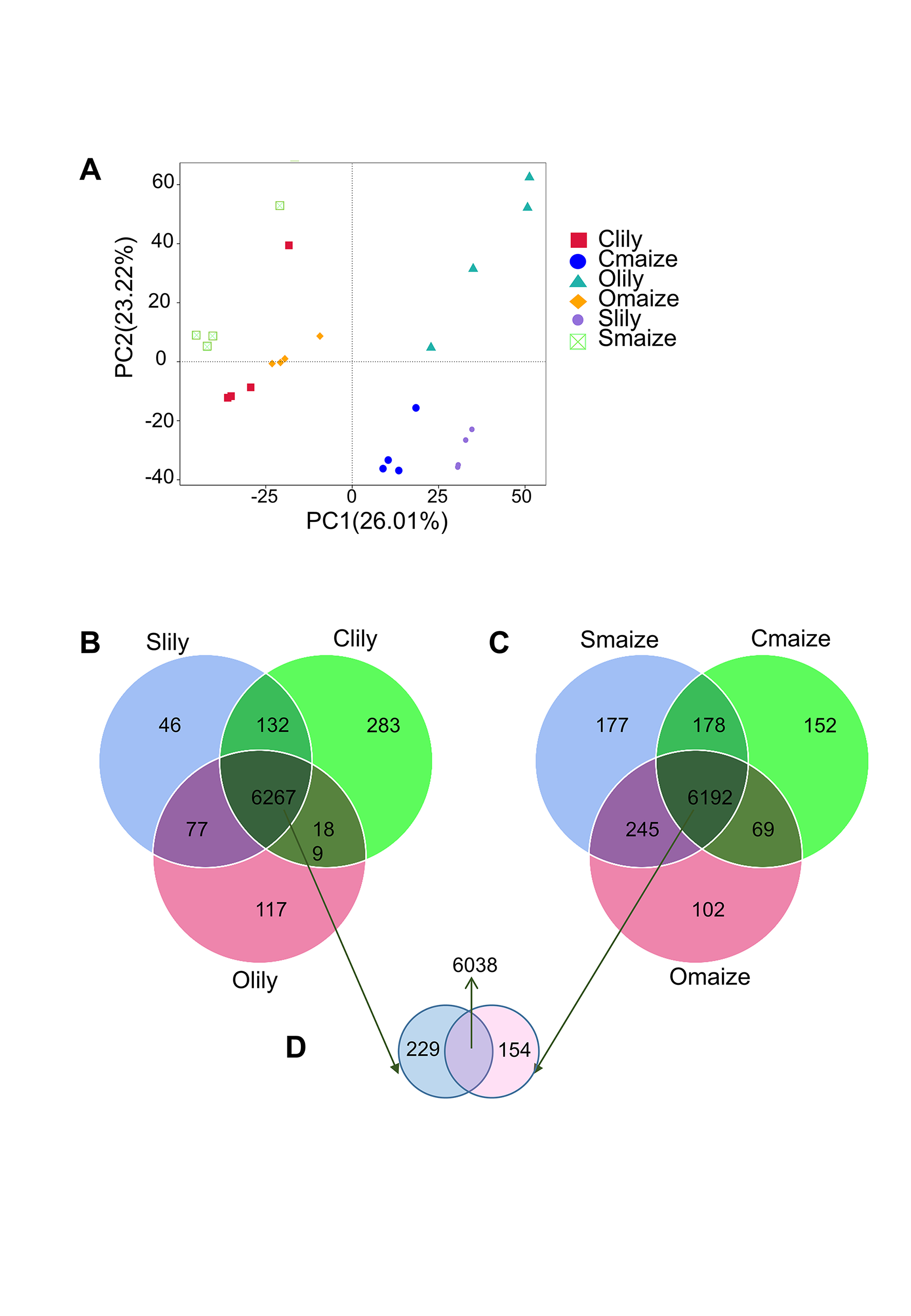

Supplement: Supplementary Figure 4 — Overall profile of the predicted functions of tiger lily- and maize-changed bacterial communities (cf. Figure 4). (A) Principal component analysis (PCA) assay of predicted microbial function indicates that the functions of maize and tiger lily bacteria communities are separate from each other across the three geological soil types. KEGG Orthology database was used for the microbial function prediction. n = 4 biological replicates. (B) Shared and uniquely tiger lily–associated ASV KO items among all soil types. (C) Shared and uniquely maize-associated ASV KO items among all soil types. (D) Shared and unique KEGG Orthology (KO) items of tiger lily and maize. [file Image_4.tif]

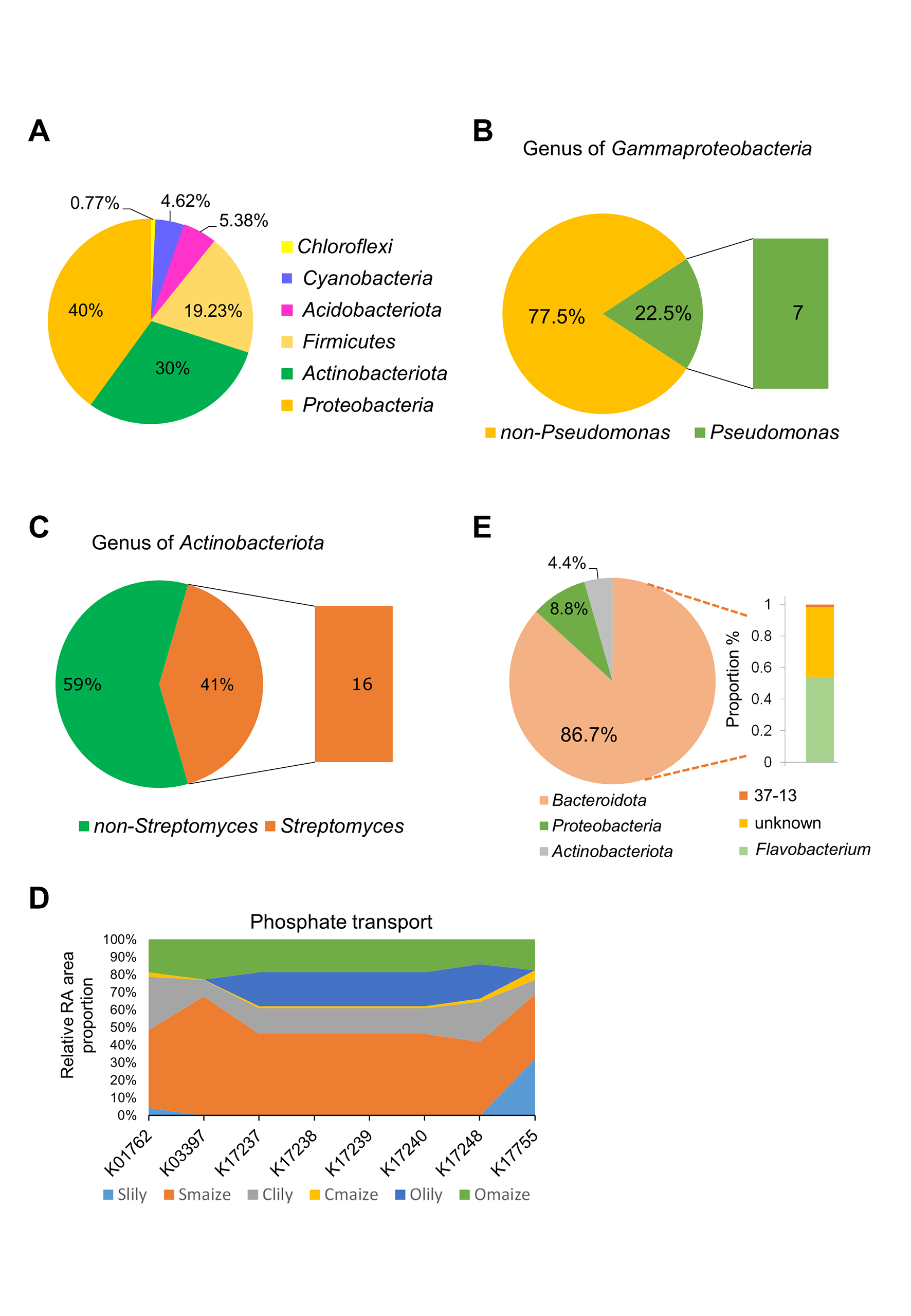

Supplement: Supplementary Figure 5 — Pseudomonas and Streptomyces are the most abundant genera in bacterial communities with pathogenic function associated with tiger lily (cf. Figure 4). (A) Distribution of the bacteria of 23 KEGG Orthologys (KOs) at the phylum level. (B) Percentage relative abundance (RA) of the Pseudomonas genus within the Gammaproteobacteria class. The number in the bar panel is the amplicon sequence variant (ASV) number of Pseudomonas. (C) Percentage RA of the Streptomyces genus within the Actinobacteria phyla. The number in the bar panel is the ASV number of Streptomyces. (D) All conserved phosphate transport function categories across the different samples. Relative RA area indicates the relative occupation of each sample compared to all samples. The larger the area of a sample, the stronger the phosphate transport ability in that sample. (E) Distribution of the bacteria of 8 KOs at the phylum level. The bar stack indicates the proportion of bacteria at the genus level within the Bacteroidota phylum. [file Image_5.tif]
